# Social Wellbeing, Board-Gender Diversity, and Financial Performance: Evidence From Chinese Fintech Companies

**DOI:** 10.3389/fpsyg.2022.862897

**Published:** 2022-05-03

**Authors:** Shahzad Ghafoor, Kwame Asare Duffour, Uzair Farooq Khan, Muhammad Kaleem Khan

**Affiliations:** ^1^School of Business Administration, Zhejiang Gongshang University, Hangzhou, China; ^2^Management Studies Department, Kumasi Technical University, Kumasi, Ghana; ^3^School of Business, Asia-Australia Business College, Liaoning University, Shenyang, China

**Keywords:** corporate social performance, financial technologies, board gender diversity, E-commerce, institutional theory

## Abstract

The main goal of this study is to investigate the relationship between female representation on board of directors and financial performance, using evidence from Chinese fintech companies, which are providing financial services using cutting-edge technologies. This research used fintech firms listed on the Shanghai and Shenzen Stock Exchange from 2010 to 2019 to test the research questions using regression analyses by SPSS v26. According to the data, the Blau index has a large and negative influence on Tobin's Q, which demonstrates that gender diversity harms the performance of Chinese enterprises. Furthermore, corporate social responsibility (CSR) is found to have a significant and positive moderating influence on Tobin's Q, implying that the adoption of CSR converts a negative to a positive effect. Diversified board members can improve the organization's control and monitoring operations, and female-represented board of directors can participate in the CSR activities that contribute to the organization's performance. The study contributes to the research of gender diversity by providing evidence that women on board of directors enhance firm performance, and the moderating function of CSR is examined with the link of female participation on the board of directors and financial performance.

## Introduction

Gender diversity is fast becoming an increasing concern in today's business environment. According to The World Post's Matt Sheehan, Jack Ma remarked that women executives constitute Alibaba's special sauce; nearly 40% of all the workers are women, including the Chief Executive Officer (CEO), Chief Financial Officer (CFO), and Chief Procurement Officer (CPO). It is all about gender equality. Women account for approximately 34% of the company's high-level managers and one-third of its founders, resulting in a balanced leadership team at Alibaba. It is all about gender equality. According to the study, Asian women are still underrepresented on corporate boards, owing to family obligations or responsibilities outside of work as well as workplace cultures that do not aggressively promote gender diversity and equality (Adams and Ferreira, 2009). According to the Deloitte Southeast Asia Center for Corporate Governance Leader, the number of women on board seats in Asia (7.8%) is increasing, although the rate of change remains moderate when compared to worldwide figures (14.5% in North America and 22.6% in Europe) (Ahmad et al., 2015). China's sophisticated integrated platforms, for example, the use of a QR code to simplify digital payments. According to studies, mobile phones might result in a 30% increase in consumers using financial services (McKinsey Global Institute, 2017). Despite the apparent financial justification for a level-playing field, discriminatory laws, such as the absence of formal property rights, mean that women are unlikely to have the collateral required for typical company financing. According to the World Bank, formal women-owned SMEs face a $300 billion annual credit shortage, with 70% of them unable to get critical funding (World Bank, 2017). An increasing amount of evidence shows that having more women in leadership roles improves corporate performance. In a study of 21,980 companies in 91 countries (World Bank, 2017), the Peterson Institute for International Economics showed that a 30% female presence on the board of directors may boost the net margin by up to six percentage points. According to Catalyst's study findings, the organizations with more women on their board of directors had a 66% greater return on investment than those with fewer women. Women join the workforce with lofty goals. According to EY, during their first 2 years in the job, 43% of the women (compared to 34% of the men) want to be in senior management roles, but this drops to just 16% after 5–7 years (compared to men staying the same at 34%). It is apparent that establishing a clear pipeline for women throughout their careers, especially as they traverse the workforce throughout their child-bearing years, is just as critical as securing leadership roles for women.

Fintech (financial technology) is transforming the financial sector at a breakneck speed (Demirguc-Kunt et al., 2018; Goldstein et al., 2019; Hau et al., 2019). Many changes, most of which were unfathomable even a decade ago, are occurring at an unprecedented scale in practically every element of China's financial industry because of digital technology (Xiao et al., 2017). China's fintech industry differs from that of several industrialized nations in numerous respects. One, in North America and Western Europe, cryptocurrencies and cross-border payment get a lot of attention in the fintech field, but in China, mobile payment, online lending, and online investing receive a majority of the headline news. Two, a limited handful of unicorn firms, such as Ant Financial, Tencent, Baidu, and JD Digits, dominate China's fintech sector. While China's fintech industry has made significant progress in terms of financial efficiency and financial inclusion, it has also caused uncertainty, financial concerns, and even societal issues. The fintech industry makes financial services more accessible and efficient. It is a worldwide problem to provide ethical and sustainable financial services to SMEs and low-income people (Nanda and Kaur, 2016). Thanks to digital technology, hundreds of millions of people, particularly SMEs and low-income families, can make mobile payments, apply for online loans, acquire digital insurance goods, and create online investment products. These are made feasible by the fintech sector's digitally reduced barriers to client acquisition and risk assessment (Xie et al., 2016). By dramatically increasing the risk management procedures, it also advances the “border” of loan availability to enterprises with poor creditworthiness (Hau et al., 2018). Leong et al. (2017) investigate the growth of a fintech business in China that provides microloans to college students, arguing that fintech may provide enterprises with a strategic edge in China's financial industry.

### Problem Statement

The percentage of women on board of directors is often lower than that of men since men have traditionally been the decision-makers from ancient times to the present. According to studies, women continue to be underrepresented in top leadership roles in American firms, resulting in the “glass ceiling,” which provides invisible hurdles to women's advancement to senior management and board positions (Chin, 2016; Chia, 2017). A more gender-diversified board might be detrimental to a company's success owing to over-monitoring. Unconscious gender prejudice has become a barrier to the advancement of women as leaders in politics and business. Unconscious biases in how people perceive and judge women create unseen hurdles for women to join and advance in the leadership pipeline, particularly on corporate boards. The diversity inherent in a board's makeup might be termed “board diversity”. Gender, age, ethnicity, country, educational background, industrial experience, and organizational membership may all be used to quantify diversity (Campbell and Mánguez-Vera, 2008). Board composition is a hot subject in corporate governance research, particularly in light of the global financial crisis (Abatecola et al., 2013), since it influences the board's effectiveness, the way it executes its functions, and, as a result, a company's financial success. Traditionally, studies have looked at the percentage of insiders on the board (Agrawal and Knoeber, 1996), the tenure of directors and managers (Hermalin and Weisbach, 1991), the share ownership of the board members (Weisbach, 1988), the size of the board of directors (Kini et al., 1995), and the type of remuneration scheme used to determine the impact of board composition on firm financial performance. However, in recent years, researchers (Mensi-Klarbach, 2014; Ntim, 2015) have started to study whether board diversity improves board effectiveness and, as a result, business financial success (Mensi-Klarbach, 2014; Ntim, 2015). Several studies have looked into the impact of women in the boardroom on a firm's financial performance (Nielsen and Huse, 2010; Cook and Glass, 2014), and whether companies with women actively involved in the boardroom outperform those with less gender diversity (Nielsen and Huse, 2010; Cook and Glass, 2014). By studying the link between female participation on board of directors and financial performance using data from Chinese publicly listed companies, we fill a gap in the research.

The major focus of the research is to investigate the association of women's representation in the board of directors with financial growth while drawing empirical evidence from the Chinese public-listed companies. The study revolves around the following objectives: To evaluate the importance of gender diversity in terms of the executive board in the contemporary business environment; to inspect, in the Chinese context, the relationship between female participation in the top management as well as its effect on the monetary output of the organization; to examine the moderating influence of corporate social responsibility (CSR) on the connection between women's representation on board of directors and monetary production using information from Chinese publicly traded businesses. First, this study analyzes the impact of female board presence on business financial performance and elucidates a significant variable in the corporate governance literature that has received little attention. Second, the study will provide support in terms of bridging the gap through the provision of information to the policymakers in China that will help in bringing improvement in trade-related policies. Moreover, the findings of the projected study will help establish the level of awareness concerning the promotion of females on the board of directors. The study is not just dynamic from the financial as well as economic perspectives, but even significant when considering the social and cultural viewpoints.

### Theoretical Review and Hypotheses Development

The Ant Group, a subsidiary of Alibaba, and Tencent, a subsidiary of Tencent, control China's massive digital financial business. Their two systems, Ant Group's Alipay and Tencent's WeChat Pay, together account for more than 90% of the third-party mobile payment business, which was valued at more than RMB 340 trillion (about US $50 trillion) in 2019. Their meteoric rise is the result of two private Chinese companies leapfrogging the traditional card-based banking system to serve a new, rapidly growing market of small and micro businesses (SMBs) and consumers seeking more convenient payment methods, increased access to credit, and better wealth management. The amazing growth of mobile payments, both in terms of volume and penetration, is due to these two companies' world-class technologies and substantial subsidies. As long as you use Alipay, we will repay you in the event of account theft, “Alpay's tagline at the time said”. Taobao's confidence as an online payment system improved, and the two platforms aided one another's growth in a positive feedback cycle. Alipay Taobao helped overseas rival eBay EachNet overtake China in 2006 with 67% of the market share compared to eBay EachNet's 29%. By August 2007, Alipay had well over 50 million users, while credit card users numbered just 30 million at the time. It has eclipsed Paypal, the US online payment counterpart in terms of transaction volume by 2014. Given the nascence of the business, research on fintech firms is a relatively young topic of academic investigation. Nonetheless, this paper contributes to a burgeoning field of interest to academics and policymakers alike, as it considers the technological, macroeconomic, financial (Frost et al., 2019; Gambacorta et al., 2019; Hassan et al., 2019; Hong et al., 2020), and monetary fields (Tucker, 2017; Eichengreen, 2019; Auer et al., 2020; Chorzempa, 2021).

Several researchers looked at the trends and impact of changes in the diversity of the workforce (Sarhan et al., 2019). The women's participation in top management significantly influences the company's overall performance (Robinson, 2008). Conversely, it is also claimed that the assortment at the top management can lead to conflicting situations, less group cohesion, lower quality and performance, and enhanced employee turnover, thus, leading to a decline in business value (Herring, 2009). Several attributes of the board members have been attributed to the above claims about the influence of women's participation presence on top management, on the performance of the organization, their cultural background, educational levels (Kusumastuti and Supatmi Sastra, 2007), their multiple directorships, remuneration type, and the degree of share ownership (Campbell and Mánguez-Vera, 2008). These characteristics influence the firm performance (Carter et al., 2003). For instance, previous research studies have been conducted in established states, such as Australia, the United Kingdom, and the United States, to identify the influence of the board members' gender on the overall board performance (Raymond et al., 2010). The financial scenario for a gender-diversity board is that it may result in a company's achieving success, and many boards have exceptional characteristics that add value to the interests of the stockholders (Carter et al., 2003). Furthermore, Madhani (2017) reinforced that the female representation on boards around the globe is far less than that of men on board.

Conflicts between the managers and agents are the main objectives of agent theory. The agency theory gives room to the board of directors to supervise and manage managers. This theory also allows the management to supervise and manage managers (Arslan et al., 2010) and to elucidate agency problems (Reguera-Alvarado et al., 2015). A significant and positive impact of gender diversity on the company's board of directors is explained by the agency theory. Gender diversity is one of the most important corporate governance mechanisms in the perspective of agency theory (Gallego-Álvarez et al., 2010). This system provides better control over the board of directors with gender diversity because different points of view and opinions can increase the independence of the board (Reguera-Alvarado et al., 2015). Thus, gender diversity in boards can be a cost-saving mechanism related to institutional issues (Reguera-Alvarado et al., 2015). The previous literature on the agency theory supposes that resilient corporate governance can reduce agency difficulties (Liu et al., 2013) and improve board oversight (Isidro and Sobral, 2014). The executive board is a critical component of the overall corporate governance structure. It is the agency responsible for the complete procedure and tactical way and is the driving force behind the performance of the firm. It is a source of insights and guidance for its stakeholders. The features of the board and the composition of the board, including, for instance, the board numbers and the diversity of boards in terms of organizational membership, ethnicity, industrial experience, age, educational background, nationality, and gender, may influence the performance of the board (Campbell and Mánguez-Vera, 2008). For our proposed study, the board members' gender diversity will be used to categorize the influence of gender on organizational performance (Fanto et al., 2011). Research suggests that firm performance involves both non-financial performance and financial performance. Tobin's Q was utilized to measure organizational financial performance.

### The Relationship Between Gender Diversity in the Boardroom and Firm Financial Performance

Several researchers looked at the trends and impacts of changes in the diversity of the workforce, particularly at the top management level, on business performance (Singh et al., 2008; Sarhan et al., 2019). Women's participation in top management is expected to have a positive impact on the firm's performance (Robinson, 2008). However, it is also argued that diversity at the top management level can lead to greater conflict, lower group cohesion, increased employee absenteeism and turnover, and lower quality and performance, thus, leading to a decline in the value of the business (Herring, 2009). Several attributes of the board members have been attributed to the above arguments on the impact of women on the performance of top management. The attributes that have an impact on the ability of the board members to perform their work effectively include their skills and abilities (Carter et al., 2007), their educational and cultural background (Kusumastuti and Supatmi Sastra, 2007), their potential involvement in multiple directorships, the level of share ownership, and the type of remuneration (Campbell and Mánguez-Vera, 2008). These attributes have an impact on the firm's performance (Carter et al., 2003).

Farrell and Hersch (2005) theorized that if women are in short supply on corporate boards, they will opt to serve on the boards of companies that perform better. Although it is conceivable that the two variables are endogenously determined, this implies a favorable relationship between the participation of women on the boards and business financial success. Other studies, on the other hand, suggest that increasing gender diversity may harm a company's financial success. Members of homogeneous groups communicate more often because they are more likely to hold the same viewpoints according to Earley and Mosakowski (2000), while Williams and O'Reilly (1998) found that homogeneous groups are more cooperative and have fewer emotional conflicts. As a result, although increasing gender diversity among the board members creates more viewpoints and critical issues (and hence more conflicts), decision-making will take longer and be less effective (Lau and Murnighan, 1998), because markets want speedy replies (Hambrick et al., 1996). Furthermore, Jianakoplos and Bernasek (1998) suggested that women are less risk-averse than men, while asserted that women raise a firm's expenses through increased turnover and absenteeism.

According to Adams and Ferreira (2009), gender diversity has a negative relationship with both Tobin's Q and ROA. Gender diversity improves performance in businesses with lower board-size protection, but it has been observed that increased supervision (such as that exercised by diverse boards) has a detrimental effect on well-governed corporations. Similarly, according to Anderson et al. (2011), the influence of diversity on a business' financial performance varies depending on company characteristics, with more complicated firms benefiting from board diversity while less complex companies suffer. In their analysis of the relationship between female directors and financial performance in the United States, Dobbin and Jung (2011) discovered a negative link between gender board diversity and Tobin's Q, but no significant relationship between gender diversity and ROA. Alternatively, Erhardt et al. (2003) looked at 112 major US companies and discovered a link between ROA and board diversity. Furthermore, Ahern and Dittmar (2012) revealed that firms with all-male boards have a negative influence on their valuation, but those with at least one female director have a favorable market response. Given the volatility of previous research findings, the subject of gender diversity and its relationship to business financial success has to be investigated further. Based on the above empirical evidence, we propose the following hypothesis:

*H1: Gender diversity in the boardroom (as measured by the Blau index) has a positive relationship with a firm's financial performance*.
*H1.1: Gender diversity in the boardroom (as measured by the Blau index) has a negative relationship with a firm's financial performance*.

### The Moderating Role of CSR

Maqbool and Zameer (2018) have defined CSR as a concept in which companies integrate social and environmental concerns into the operations of the business, which overall leads to interaction among the board sizes voluntarily. CSR has become increasingly important in the current era for organizations as it supports enhancing the competitiveness of the firms. According to the stakeholders and agency theory, CSR is considered to be an important aspect for the enhancement of financial performance. Several studies have been investigated to evaluate the influence of CSR on financial performance (Galant and Cadez, 2017; Oyewumi et al., 2018; Al-Hadi et al., 2019). A study conducted by Akben-Selcuk (2019) investigated the influence of CSR on a firm's performance of the developing country, Turkey, where the data were gathered from the Borsa Istanbul (BIST)-100 index from 2014 to 2018. The empirical evidence through using the regression analysis has illustrated that CSR has a significant and positive influence on financial performance. In addition, Boubaker et al. (2020) have also indicated that the activities of CSR help in reducing the financial distress risk, which means that it often saves the company from bankruptcy due to gaining support from the stakeholders, particularly the investors, board members, government, and others. Opinions in the previous literature confirm the positive impact of female managers on a firm's performance. First, diversified management means that diversified management can increase a company's profitability and value by bringing distinctive potential, skills, and talents to the board (Carter et al., 2008). Also, gender diversity can improve problem-solving skills by incorporating a variety of evaluations into board discussions (Campbell and Mánguez-Vera, 2008). In this respect, different perspectives may offer alternatives to decision-makers and allow them to reflect more carefully (Carter et al., 2003). Board of directors with diverse skills, cultural backgrounds, and genders offer strategic resources for improving firm performance (Ujunwa et al., 2012).

Following the study conducted by Yasser et al. (2017), CSR requires the firms to utilize their funds and resources for social improvement that leads to promoting the goodwill of the company. CSR has been determined to have a positive effect on the overall performance of the organization, in that, it enables the organization to gain access to valuable resources, attract and retain higher-skilled employees, develop opportunities, and contribute toward social legitimacy. Al Fadli et al. (2019) have illustrated that diversified board members are likely to enhance the control and monitoring of the activities of the organization, along with the improvement of the disclosure practices that overall improve the interests of the board-size groups. The diversified board of directors is also expected to be engaged in CSR activities and reporting, which can help in reporting corporate transparency, voluntary information, and facilitating better engagement among the board-size group. The enhancement of the CSR activities as a result of gender diversity overall enhances the financial performance. There are a wide number of studies that have been conducted to evaluate the moderating role of CSR on gender diversity and financial performance (Arujunan et al., 2018; Jiang et al., 2020; Saleh et al., 2021). The study conducted by Jiang et al. (2020) was to evaluate the moderating role of CSR on gender diversity and a firm's financial performance in the emerging economy. Hence, the following hypothesis is developed as per the conceptual framework:

*H1.2: CSR significantly and positively moderates gender diversity and financial performance*.
*H1.3: CSR significantly and negatively moderates gender diversity and financial performance*.

## Methodology

### Research Design and Data Collection

The present study used a quantitative approach to examine the relationship between gender diversity in boardrooms and firm financial performance. The samples used in this study comprise all financial firms listed on the Shanghai (SH) and Shenzen (SZ) Stock Exchanges. The financial firms consisted of banks, insurance companies, security companies, financial institutions, and other types of financial firms. The reason for selecting the financial companies' data is that all fintech companies provide facilities utilizing the internet and various information technology devices. The data consisted of companies in the years 2010–2019, which makes a time frame of 10 years. The panel or longitudinal study was adopted since the data were collected over a period and summarized statistically (Hair et al., 2006). Data analysis was conducted using SPSS v.26, where the mean, frequencies, and other tests were used to analyze the data. Pearson's linear correlation coefficient and regression analysis were used to find the relationship between the variables. A bootstrapping method was conducted for the significant investigations of the research hypotheses.

### Measurement of Variables

#### Tobin's Q

Tobin's Q was used to approximate the organization's market-oriented monetary production. Such a metric is commonly referred to as the marketplace or value of the firm or the worth of the company and this description was used in the research. Previous studies have used this measure to assess a firm's market-oriented economic health (Hillman and Cannella, 2007; Moreno-Gómez et al., 2018). It has been extensively used in research that has investigated the relationship between female members and a company's monetary production. Tobin's Q was designed to calculate the total book value of debt as well as the share price times the overall numbers concerning the outstanding shares, with the division through the book value of the overall assets. Tobin's Q was calculated by summing the book value of total debt and the share price times the total number of outstanding shares and dividing by the book value of total assets. Following from Campbell and Vera (2008), Tobin's Q is calculated as follows:


$$\begin{array}{l} \text{Tobi}{\text{n}}^{\prime }\text{sQ = }[\text{Book value oftotal debt }\\ +(\text{share price x total numbers of outstanding shares})]\;\\ \text{/Book value of total assets} \end{array}$$

#### Blau Index

The independent variable used in this study was gender diversity in the boardroom. The Blau index was believed to be appropriate to be used in the study to measure gender diversity in the boardroom.


$$\begin{array}{l} \text{Blau index}=1-\sum \text{\_limits\_}{\left\{\text{i}=1\right\}}^{\text{k}}\left\{\text{p\_}{\text{i}}^{2}\;\right\}, \end{array}$$

Where *p* is the proportion of the group members in the ith category and *n* is the total number of board members. The variables used in the conceptual framework are presented in Table 1.

**Table 1:** Measurement of constructs.

**Sr. No**	**Variables**	**Type**	**Measures**	**Source(s)**
1.	Financial Performance	Dependent	- Tobin's Q	Annual reports of sample firms, downloadable from the website of the Shanghai (SH) http://english.sse.com.cn/home/ and Shenzen Stock Exchange (SZ) https://www.szse.cn/English/
4.	Gender Diversity	Independent (variable of interest)	Blau index	Annual reports of sample firms, in the section of: - Biography and background of Board Members - Notes to Financial Statement
5.	Firm Size	Control	The natural logarithm of total assets	Annual reports of sample firms, downloadable from the website of the Shanghai (SH) http://english.sse.com.cn/home/ and Shenzen Stock Exchange (SZ) https://www.szse.cn/English/
6.	Debt level or Leverage	Control	The ratio of total debt to total assets	Annual reports of sample firms, downloadable from the website of the Shanghai (SH) http://english.sse.com.cn/home/ and Shenzen Stock Exchange (SZ) https://www.szse.cn/English/
7.	Board Size	Control	The natural logarithm of total number of board members	Annual reports of sample firms, in the section of:–Biography and background of Board Members–Notes to Financial Statements
8.	Corporate Social Responsibility (CSR)	Moderating Variable	The amount of donations made by organization	Annual reports of sample firms, downloadable from the website of the Shanghai (SH) http://english.sse.com.cn/home/ and Shenzen Stock Exchange (SZ) https://www.szse.cn/English/

#### Blau Index

The independent variable that was utilized in this research was the boardroom gender diversity ratio. Moreover, the Blau index was selected for measuring diversity because of its aptitude for utilizing two gender groups, which are men as well as women, and the distribution balance of the board members among them (Adams and Ferreira, 2007; Vera and Martin, 2011). The Blau index is usually called the proportion calculation for measuring the variability as well as determining the diversity level (Chemers et al., 1995; Pitts, 2005).

In such a case, the Blau index is a commonly identified proportion calculation for measuring the variability as well as for determining the diversity level among a set of individuals (Pitts, 2005). The Blau index is utilized in the present research through the study of Harrison et al. (2002) to measure group diversity, which involves the gender as a diversity type.


$$\begin{array}{l} \text{Blau index}=1-\sum \text{\_limits\_}{\left\{\text{i}=1\right\}}^{\text{k}}\left\{\text{p\_}{\text{i}}^{2}\right\}, \end{array}$$

In this, *p* refers to the number of the group associates in the ith group, whereas *n* is the overall number of the board members. In such a formula, the rates concerning the Blau index tend to be from zero, that is, assigned to the comparable group having the minimum value, toward the positive one, which is the supreme value when the equal distribution of every set is in a group. The highest value of the Blau index, however, varies based on the total number of categories. For two groups, men as well as women, as the groups for the diversity of genders, the greater Blau index can be 0.5 when the same men, as well as women, are in an experiential group. If the greater groups are functional, the highest rate of the Blau index becomes equivalent to each other. Thus, in the present research, when it was discovered that the Blau index reached 0.5, it meant that the group's diversity of gender may be at its maximum, whereas when the value reached 0, the group was overall similar.

#### Moderating Variable

The moderating variable that is signified in the given study is the CSR, whose role is examined in terms of gender diversity as well as the performance of the firm. The variable CSR tends to represent the provision of social amenities as well as caring for the environment and the people in the community/society in which corporations operate (Ioannou and Serafeim, 2015).

**Figure 1:**
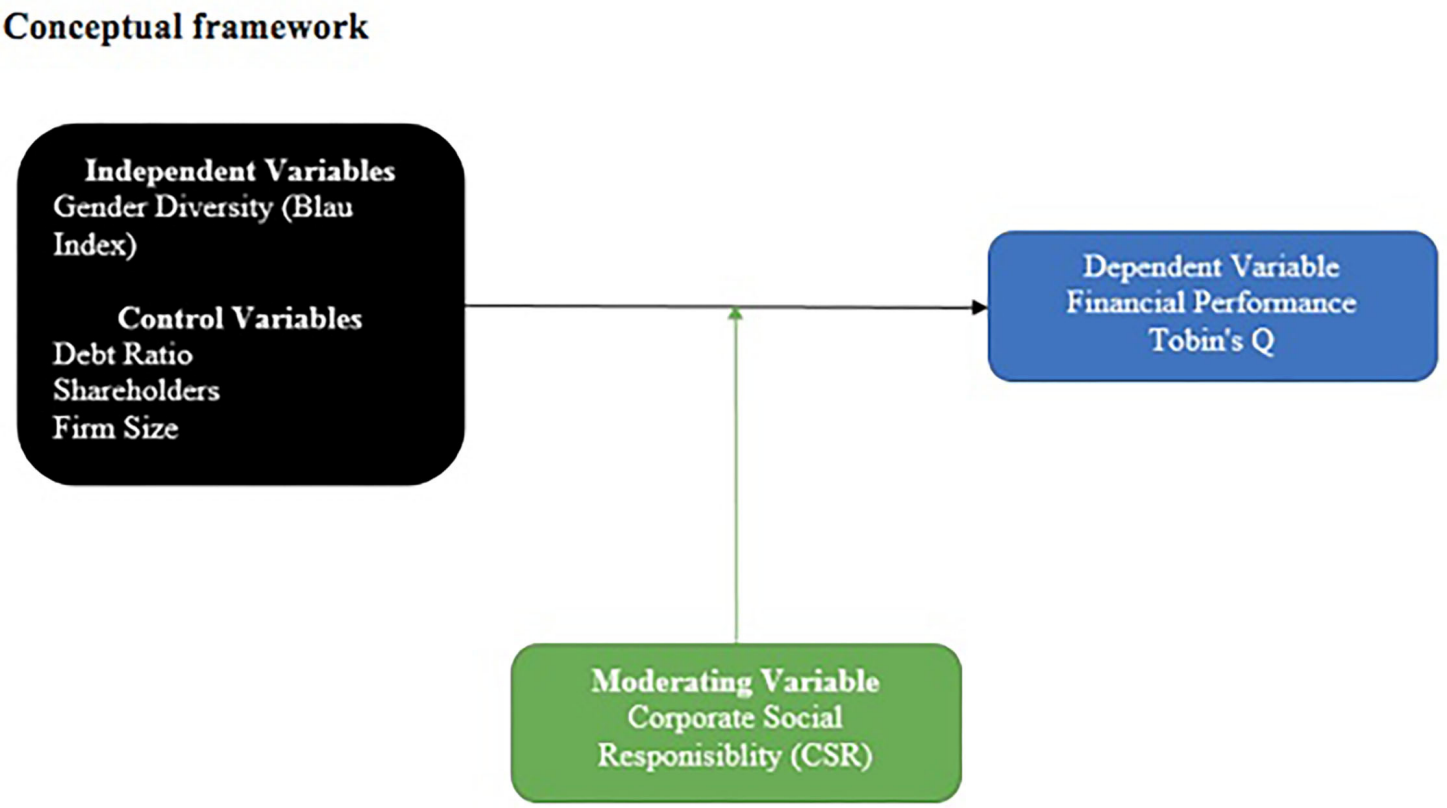
Conceptual framework. Source: Researcher's construction.

Figure 1 represents the conceptual framework which is based on the aim of the research. Tobin's Q represents the dimension of the financial performance of the organization. On the other hand, the independent variable is gender diversity that is measured with the support of the Blau index. In addition, several other control variables are incorporated into the study which are the firm's size, debt level, and board size. Furthermore, the moderating variable is also incorporated into the study which consists of CSR, where its moderating effects are examined between gender diversity and financial performance. The statistical model of the following study is provided below:


$$\begin{array}{l} Tobin^{{}^{\prime }}s\;Q=a+\beta_{1}GD+\beta_{2}DR+\beta_{3}SH+\beta_{4}FS+\beta_{5}CSR\\ \;+\beta_{6}GD\;*\;CSR+\varepsilon \end{array}$$

## Results

### Descriptive Statistics

Table 2 elaborates the descriptive statistics of the variables. It represents the mean value of the Blau index which is 0.96 with a standard deviation of 0.05. This indicates that the average diversity index of the sample companies is 0.96 that is deviated by 0.05 above or below. Tobin's Q has the mean value of 2.40 with a standard deviation of 13.88 which shows that the average organization value of the data is 2.40 that can deviate by 13.88 above or below. The debt ratio has been identified to have a mean value of 0.50 with a standard deviation of 15.15. This implies that the average debt ratio is 0.50 which might deviate by 15.15 above or below. The firm size has a mean value of 21.98 with a standard deviation of 1.46 which reflects that the average firm size of the sample data is 21.98 that can be deviated by 1.46 above or below average. The CSR has a mean value of 25.46 with a standard deviation of 17.06, which shows that the CSR in the observed companies is an average of 25.46 that can be deviated by 17.06 above or below. The board size has a mean value of 13.99 with a standard deviation of 6.45, which shows that the board-size average in the given sample is 13.99 with a standard deviation of 6.45.

**Table 2:** Descriptive statistics.

**Variable**	**Obs**	**Mean**	**Std. Dev**.	**Min**	**Max**
Blau Index	23,941	0.96	0.05	0.56	1
Tobin's Q	23,941	2.40	13.88	0.15	1,789.7
Debt Ratio	23,940	0.50	15.15	−0.2	2,342.97
Firm Size	23,940	21.98	1.46	13.37	30.9632
CSR	23,941	25.46	17.06	−18.46	90.87
Board size	23,941	13.99	6.45	−13.12	28.19

### Correlation Analysis

Table 3 represents the correlation analysis of the variables. It indicates that Tobin's Q is significantly related to Blaus index, debt ratio, firm size, CSR, and board size. The Blau index, firm size, CSR, and board size have a negative and significant relationship with the Tobin's Q having r = −0.02; r = −0.13; r = −0.03, and r = 0.04, respectively. The debt ratio has been identified to have a positive relationship with Tobin's Q having r = 0.06. Based on these associations, it can be inferred that the relationship of firm performance measured by Tobin's Q is weak with the other variables since all the values are below 0.3. In addition, the association is computed to be statistically significant at 5% which indicates that the *p*-value of the association is below 5% or 0.05.

**Table 3:** Correlation analysis.

	**Tobin's Q**	**Blau Index**	**Debt Ratio**	**Firm Size**	**CSR**	**board size**
Blau Index	−0.02^**^	1				
Debt Ratio	0.06^**^	0.005	1			
Firm Size	−0.13^**^	0.0688^*^	−0.03^*^	1		
CSR	−0.03^**^	0.0396^*^	−0.01^*^	0.33^*^	1	
board size	−0.04^**^	0.0433^*^	−0.03^*^	0.16^*^	0.57^*^	1

** *significant at 5%*;

* *at 10%*.

### Robustness Check

Based on the earlier research in the CSR literature (Margolis et al., 2007; Wang et al., 2015), our study used ROE as a financial success metric. Return on assets (as a supplementary accounting measure), or ROA, and the market-to-book ratio (M/B), as a market-based measure of business performance, are analyzed to assess the robustness of the results. ROA is calculated by dividing the net income by the total assets in 2019. For 2019, the M/B is calculated by dividing the year-end market value of a company's common stock plus the book value of its preferred stock and the debt by the book value of its total assets.

### Regression Analysis

Panel data regression has been used in which the random effect model has been determined and then the fixed-effect model has been applied for regression analysis. Based on the Hausman specification, the suitability of random effect and fixed-effect data have been used. Hausman has the null hypothesis that the random effect model is applicable and alternative hypothesis that the fixed-effect model is applicable.

Table 4 represents the regression analysis of the model when the dependent variable is Tobin's Q. The Hausman specification test indicates a *p*-value of 0.00, and hence, the null hypothesis is rejected and the fixed-effect model is applied. Spearman's multicollinearity has been tested and it is elaborated that the variables do not have the multicollinearity issue as illustrated in Table 5. The Blau index has been found to have a significant and negative influence on Tobin's Q with a *p*-value of 0.002. The debt ratio has also been found to have a significant and positive influence with a *p*-value of 0.00. The firm size has a *p*-value of 0.00, and hence, indicates a significant and negative influence on Tobin's Q. The CSR also shows a significant and negative influence with a *p*-value of 0.05. However, the board size has been found to have an insignificant and negative influence on Tobin's Q with a *p*-value of 0.12.

**Table 4:** Regression analysis.

	**Random effect**		**Fixed effect**	
**Tobin's Q**	**Coefficient**	***P*-value**	**Coefficient**	***P*-value**
Blau Index	−4.28^**^	0.02	−6.45^***^	0.002
Debt Ratio	0.05^***^	0.00	0.05^***^	0.000
Firm Size	−1.39^***^	0.00	−2.55^***^	0.000
CSR	0.02^**^	0.02	−0.01^*^	0.050
Board size	−0.07^***^	0.00	−0.03	0.120
_cons	37.60^***^	0.00	65.57^***^	0.000
Hausman	0.00			

*** *significant at 1%*;

** *at 5%*;

* *at 10%*.

**Table 5:** Spearmen's matrix.

	**Tobin's Q**	**Blaus index**	**Debt ratio**	**Firm size**	**CSR**	**Board size**
Blaus index	−0.03^*^	1				
Debt ratio	−0.29^*^	−0.01	1			
Firm size	−0.55^*^	0.03^*^	0.45^*^	1		
CSR	−0.13^*^	0.03^*^	−0.08^*^	0.27^*^	1	
Board size	−0.04^*^	0.05^*^	−0.27^*^	0.13^*^	0.74^*^	1

* *significant at 10%*.

### The Moderating Effect of CSR

To determine the moderating effect of CSR, the researcher computed the interaction effect between CSR and Blau index, which is the main independent variable of the study representing the female ratio. In this regard, the fixed effects model has been tested, which has been presented in Table 6. Following the results, the overall variance explanation of the model is 2.02%, which is extremely minimal. This implies that the variance in Blau index, debt ratio, firm size, board size, CSR, and the moderating effect of CSR explains 2.02% of the variance in the firm performance concerning Chinese firms, which is indicated and measured by Tobin's Q. In terms of the overall significance of the model, it has been found that the f-statistics is computed to be 78.270 while the *p*-value is computed to be 0.000. This implies that the overall model is statistically significant since the *p*-value is below 5%, or 0.05.

**Table 6:** Moderating effect of CSR.

**Tobin's Q**	**Coef**.	**Std**.	***t***	***P* > |t|**
Blau's index	−12.606^***^	3.371	−3.740	0.000
Debt ratio	0.046^***^	0.006	8.440	0.000
Firm size	−2.547^***^	0.137	−18.550	0.000
Board size	−0.032	0.022	−1.470	0.142
CSR	−0.266^**^	0.109	−2.450	0.014
CSR*Blau's index	0.260^**^	0.112	2.320	0.021
Constant	71.328^***^	4.403	16.200	0.000
f-statistics	78.270		R-squared	2.02%
*P*-value	0.000			

*** *significant at 1%*;

** *at 5%*.

On the other hand, to determine the individual significance of each variable, the results indicate that Blau index is statistically significant. The coefficient value is computed to be B =-12.606, which is significant at 1% (*p* = 0.000–0.01). The negative sign implies that an increase in female or female representation in Chinese firms would lead to lower firm performance as indicated by Tobin's Q. In addition, the effect of the debt ratio is computed to be statistically positive with B = 0.046 and *p* = 0.0001–0.01. This implies that high gearing in Chinese firms can lead to a better Tobin's Q or firm performance. Besides, the effect of firm size is computed to be negative with B = −2.547, which is statistically significant as well (*p* = 0.000– 0.01). The negative sign indicates that the larger the firm, the lower the performance is related to the Chinese firms. However, the effect of the board sizes is computed to be statistically insignificant since the *p* > 5%. In addition, the direct effect of the CSR is computed to be statistically negative, which is due to the presence of an interaction effect. Nonetheless, the interaction effect or moderating effect is computed to be statistically positive and significant, having a *p*-value of 0. 021, 0.05, and a beta coefficient of 0.260. This implies that in the presence of effective CSR activities, the effect of female representation turned out to be positive.

### Hypotheses Assessment Summary

In light of the results, a summary of the proposed hypotheses has been presented in Table 7.

**Table 7:** Hypotheses assessment summary.

**Hypotheses**	**Decision**
H1: Gender diversity in the boardroom (as measured by the Blau index) has a positive relationship with firm financial performance (as measured by Tobin's Q).	Rejected
H1.2: CSR significantly and positively moderates between gender diversity and financial performance (as measured by Tobin's Q)	Accepted
H1.1: Gender diversity in the boardroom (as measured by the Blau index) has a negative relationship with firm financial performance (as measured by Tobin's Q).	Accepted
H1.3: CSR significantly and negatively moderates between gender diversity and financial performance (as measured by Tobin's Q)	Rejected

## discussion

This study was conducted to identify the influence of gender diversity in the boardroom on a firm's financial performance. Gender diversity is measured by the Blau index, and firm performance is measured by Tobin's Q. The data have been collected from the annual reports of all the firms that are listed in SH and SZ publicly from 2010 to 2018. The data have been analyzed through different statistical analyses. The results of the study have identified that board-gender diversity tends to have a significant and negative influence on Tobin's Q. It has also been identified that board-gender diversity has a negative relationship with a firm's performance and have investigated the role of board-gender diversity in the financial performance of Croatian-listed firms. The study's finding supports the finding of this research since it demonstrates that board diversity tends to have a positive and significant influence on a firm's performance measured by Tobin's Q. However, this research has indicated that board diversity tends to influence a firm's performance, which contradicts the finding of this study. Boubaker et al. (2020) have evaluated the influence of board-gender diversity on the financial performance of the firms that are listed on the French stock exchange. The study has indicated that board-gender diversity does not statistically significantly affect a firm's performance as measured by Tobin's Q. This is inconsistent with the findings of this study. Mirza et al. (2012) have examined the role of board-gender diversity in the financial performance of the firms measured by the Blau index. The study has been conducted in the context of Pakistan. It has been found that board-gender diversity harms a firm's performance. The study has been inconsistent with the finding of this study, which found a negative and insignificant influence of board-gender diversity on a firm's performance and have indicated that a lack of women's representation on the board of directors has been an important issue that needs to be addressed because of the benefits that are driven by board-gender diversity. The study has evaluated the role of board-gender diversity on the firm's performance of Malaysian-listed companies. The results of the study have shown that board-gender diversity tends to influence a firm's performance positively, hence contradicting the findings of this research.

## Limitations and Implications for Future Research

The findings from this study suggest that having a variety of genders in the boardroom may be important concerning the Chinese publicly listed corporations, as these non-based executives and investors may give more of a global focus as well as larger equity portfolios that these firms often possess. The study also has significant implications for governments and legislators (market regulators) on the one hand, and board sizes and business leaders on the other hand. Both groups should carefully study our findings to strengthen government policies and business decisions that attract women to Congress. In practice, this research aids firms in better understanding the dynamism of CSR and better implementation. This study has ramifications for establishing female quotas on corporate board of directors as well. Finally, the results of this study are certainly interesting for other developing countries that have not yet adopted binding laws or disclosure guidelines/requirements to increase female participation in conference rooms. The future analysis can include other independent variables like age, experience, and education as gender is regarded as one aspect of board diversity. Further studies could reexamine the relationship between board-gender diversity in other developing countries to compare these findings.

## Conclusion

This research demonstrates the importance of female representation on the board of directors in Chinese public firms while also showing how CSR can interlink gender diversity and a firm's performance. The goal of this article was to provide fresh insights into the link between women on board of directors and the financial performance of companies. This shows that a correct balance between men and women, rather than merely the existence of women, should be most essential for Chinese fintech-listed firms. The purpose of this article was to provide fresh insights into the link between women on boards and corporate financial success. This implies that the priority for Chinese fintech-listed firms should be the appropriate balance of men and women, rather than merely the existence of women. While much remains to be learned about a board's composition and a firm's financial performance, the findings of this study, when considered alongside other recent research, suggest that board-gender diversity is not a simple “numbers game” (Post and Byron, 2015); greater gender diversity may generate economic gains; greater gender diversity does not destroy board-size value, but may effectively influence the investors' evaluations of future earning potential; and greater gender diversity does not destroy board-size-value, but may effectively influence the investors' evaluations of future earning potential. Similarly, it is critical that Chinese fintech-listed firms have access to a pool of suitably skilled female applicants for boardroom posts to sustainably enhance the company's performance. Otherwise, the benefits of having women on boards are unlikely to be reflected in any of the selected performance metrics.

To summarize, the evidence for a direct association between women on the boards and financial success is, at best, inconsistent. Due to conflicting data, some researchers suggest that a straight correlation is oversimplified since there are other possible mitigating variables (Hambrick, 2007). However, less study has been conducted to assess the validity of other theories, such as those that contain mediation. To address such a research deficit, the claim is made that the relationship between women on the boards and financial success is indirect. The findings add to the theoretical debate on how women on the boards might contribute to good corporate governance while also providing insights into the processes that may relate board qualities to financial performance.

## Data Availability Statement

The article/Supplementary Material contains the original contributions given in the study; further questions should be referred to the corresponding author(s).

## Author Contributions

SG: conceptualization, methodology, formal analysis, and review and editing. KD: data curation, formal analysis, and writing, review, and editing. UK: investigation and writing, review, and editing. MK: resources and supervision. All authors contributed to the article and approved the submitted version.

## Conflict of Interest

The authors declare that the research was conducted in the absence of any commercial or financial relationships that could be construed as a potential conflict of interest.

## Publisher's Note

All claims expressed in this article are solely those of the authors and do not necessarily represent those of their affiliated organizations, or those of the publisher, the editors and the reviewers. Any product that may be evaluated in this article, or claim that may be made by its manufacturer, is not guaranteed or endorsed by the publisher.

## Appendix

### Description of Variables

**Table:**

**Variables**	**Definition**
Tobin's Q	The sum of the market value of the stock and the book value of debt divided by the book value of total assets
Blau	An index to measure gender diversity
Board size	Calculated as the logarithm of the total number of members on the board
Firm size	Calculated as the natural logarithm of total assets
Leverage	Calculated as the ratio of the total debt to total assets
